# Variability and seasonality of active transportation in USA: evidence from the 2001 NHTS

**DOI:** 10.1186/1479-5868-8-96

**Published:** 2011-09-14

**Authors:** Yong Yang, Ana V Diez Roux, C Raymond Bingham

**Affiliations:** 1 Department of Epidemiology, Center for Social Epidemiology and Population Health, University of Michigan, Ann Arbor, Michigan, USA; 2 Transportation Research Institute, University of Michigan, Ann Arbor, Michigan, USA

**Keywords:** Active transportation, seasonality, NHTS

## Abstract

**Background:**

Active transportation including walking and bicycling is an important source of physical activity. Promoting active transportation is a challenge for the fields of public health and transportation. Descriptive data on the predictors of active transportation, including seasonal patterns in active transportation in the US as a whole, is needed to inform interventions and policies.

**Methods:**

This study analyzed monthly variation in active transportation for the US using National Household Travel Survey 2001 data. For each age group of children, adolescents, adults and elderly, logistic regression models were used to identify predictors of the odds of active transportation including gender, race/ethnicity, household income level, geographical region, urbanization level, and month.

**Results:**

The probability of engaging in active transportation was generally higher for children and adolescents than for adults and the elderly. Active transportation was greater in the lower income groups (except in the elderly), was lower in the South than in other regions of the US, and was greater in areas with higher urbanization. The percentage of people using active transportation exhibited clear seasonal patterns: high during summer months and low during winter months. Children and adolescents were more sensitive to seasonality than other age groups. Women, non-Caucasians, persons with lower household income, who resided in the Midwest or Northeast, and who lived in more urbanized areas had greater seasonal variation.

**Conclusions:**

These descriptive results suggest that interventions and policies that target the promotion of active transportation need to consider socio-demographic factors and seasonality.

## Introduction

Regular physical activity is important for the health and well being of people of all ages [1]. It reduces the risk of chronic diseases and enhances mental health [2]. Active transportation including walking and bicycling is not only an important source of physical activity, but also has positive effects on climate change and air pollution [3]. Unfortunately, walking and bicycling for transportation have declined over the past few decades in the US [4]. This trend has been observed in all age groups including children and adolescents, adults and the elderly [5,6]. Promoting active transportation is a challenge for the fields of public health and transportation [7].

Environmental effects on active transportation have received increasing attention because of their relevance for policy [8-12]. Most research has focused on the built environment such as land use mix, land use density, street connectivity, and access to transportation, while the effects of seasonality and weather conditions, have been relatively neglected [13]. Humans' physical activity including active transportation, are undoubtedly influenced by seasonality [14]. People have evolved different physical activity patterns to cope with geographically varying seasonal climate changes [15]. In the short-term, changes in weather conditions such as the amount of daylight, temperature and precipitation, can impede or promote both the desire for and the feasibility of active transportation [16].

Generally, levels of physical activity are higher in spring and summer and lower in winter [13,15-19]. However this seasonal variation can be modified by geographic region as well as by demographic, cultural and social factors. For example, in contrast to the northern states, in southern states of the US where the summer months are hot and humid, children have lower physical activity in summer than in winter [20]. The impact of season may also be modified by economic and cultural factors: in developing countries opportunities for hunting and crop cultivation determine seasonal activity while temperature and rainfall are key determinants in developed countries [21]. Seasonal differences in physical activity may also vary by age and gender, for example, in Norway children were found to be more sensitive to seasonality than adolescents [22] while in the Netherlands seasonal variation was greater in males than in females [23].

Although the impact of seasonal variations on physical activity has been systematically reviewed [13,24], most studies included in these reviews were conducted in relatively small regions with little climate variation. Only a small number of studies covered the whole US [19,25-29], and differences in patterns across population subgroups were infrequently investigated [18]. Studies which cover a range of climate regions and which investigate variations across socio-demographic groups are needed to assist in the design of more effective physical activity promotion policies.

This study used 2001 data from a large national sample to describe monthly variation in active transportation in the US by selected demographic and regional factors including age, gender, race/ethnicity, household income level, geographical region and urbanization level. In addition to overall patterns, we examined seasonal variations as well as the extent to which seasonal variations differed by demographic, and regional characteristics that could be useful in planning intervention.

## Methods

The National Household Travel Survey (NHTS) 2001 http://nhts.ornl.gov/ is a survey of personal transportation in the US. The NHTS 2001 updated information gathered in prior Nationwide Personal Transportation Surveys (NPTS) conducted in 1969, 1977, 1983, 1990, and 1995. This survey was conducted by computer-aided telephone interviews from March 2001 through July 2002. The target population was the US civilian population from infancy through 88 years of age. List-assisted random-digit dialing was used to sample households. The sampling frame consisted of all telephone numbers in 100-banks of numbers in which there was at least one listed residential number. Telephone numbers were sorted according to geographic and population variables and a systematic sample was then selected from the sorted list. For the national sample, all telephone numbers in the frame of 100-banks had an equal probability of selection. The national sample was increased in several add-on areas: New York State, Wisconsin, Texas, Kentucky, Hawaii, Lancaster Pennsylvania, Baltimore Maryland, Des Moines, Ohio and Oahu Hawaii. An adult proxy was required for individuals less than 14 years old, and 14- and 15-year-olds responded for themselves if their parent approved. The survey included 160,758 people (with written informed consents) in 69,817 households and collected information on 642,292 daily trips including the purpose, transportation mode, travel time, and time of the day. For this study, data were weighted by personal weights (provided by NHTS) to adjust for the selection probabilities at the individual level.

In this study, active transportation was defined to include walking and bicycling. The population was grouped by age into four groups: children (5-10 years old, denoted by **C**), adolescents (11-17 years old, denoted by **T **for teenagers), adults (18-64 years old, denoted by **A**) and elderly (65 years and above, denoted by **E**). Respondents were also classified based on gender, race/ethnicity, household income level, region, and urbanization level. Race/ethnicity was classified as White, Black, Asian and Hispanic. Household income level was categorized as (1) less than 20,000 dollars per year; (2) 20,000-40,000; (3) 40,000-80,000; and (4) more than 80,000. The US was divided into four sections based on US Census Region: West, Midwest, Northeast and South [30]. Level of urbanization was classified as (1) rural; (2) town; (3) suburban; (4) second city, and (5) urban based on population density [31]. Of the 160,758 NHTS respondents, 30,536 were excluded because they were of race/ethnic groups too small for reliable analysis (races/ethnicities other than the four mentioned above) or because they were missing data on key variables (12,329 for household income level, 12,142 for age, 9,384 race/ethnicity, 48 urbanization level and 21 gender), leaving 130, 222 persons for analysis. Characteristics of the population used for this study were described in Table 1.

**Table 1:** Characteristics of the study population

*Age group*		5-10 years	11-17 years	18-64 years	65+ years	All
**Percentage (%)**		9.8 (n = 12723)	11.1 (n = 14442)	66.9 (n = 87053)	12.3 (n = 16004)	100

***Sex***	**Male**	51.8	51.3	49.2	42.6	48.9

	**Female**	48.2	48.7	50.8	57.4	51.1

***Race/ethnicity***	**White**	70.9	73.9	77.1	86.2	77.3

	**Black**	14.3	16.6	12.5	10.5	12.9

	**Asian**	2.6	2.1	2.9	1.1	2.5

	**Hispanic**	12.2	7.4	7.6	2.2	7.3

***Household income level***	**< 20 k**	15.7	13.0	12.6	30.6	15.2

	**20-40 k**	23.6	21.8	23.8	36.3	25.1

	**40-80 k**	36.9	39.0	38.0	24.4	36.4

	**> 80 k**	23.9	26.2	25.5	8.6	23.4

***Region***	**Northeast**	18.1	18.9	18.7	20.7	18.9

	**Midwest**	24.3	24.6	23.3	24.5	23.7

	**South**	34.7	35.3	36.2	36.5	36.0

	**West**	22.9	21.2	21.9	18.2	21.5

***Urbanization level***	**Rural**	21.8	24.0	20.5	21.6	21.1

	**Town**	24.1	23.2	22.3	21.3	22.4

	**Suburban**	23.0	24.3	24.3	22.7	24.0

	**Second city**	17.4	15.8	17.7	21.0	17.9

	**Urban**	13.8	12.7	15.2	13.3	14.6

***Month***	**January**	8.5	8.7	8.7	7.7	8.5

	**February**	8.0	7.3	7.8	7.2	7.7

	**March**	9.0	9.0	8.5	8.1	8.6

	**April**	8.4	8.2	8.2	8.0	8.2

	**May**	8.2	8.8	8.1	9.4	8.4

	**June**	8.0	8.1	8.1	8.4	8.1

	**July**	8.3	8.1	8.2	9.8	8.4

	**August**	7.8	8.0	8.4	9.7	8.5

	**September**	8.3	8.1	8.2	8.5	8.3

	**October**	8.8	8.0	9.0	7.0	8.6

	**November**	8.0	8.5	8.2	8.1	8.2

	**December**	8.8	9.2	8.6	8.1	8.6

Four variables were used to describe the monthly variation of transportation: (1) the mean number of all trips per person per day; (2) the mean number of active trips per person per day; (3) the percentage of people who take at least one active trip in a day; and (4) the percentage of active trips amongst all trips less than one mile. Subsequent analyses focused on percentage of people who take at least one active trip in a day (denoted by PAT), because the percent of active trips among all trips was unstable due to small numbers of daily trips among some individuals. For each age group, logistic regression was used to identify predictors of PAT including gender, race/ethnicity, household income level, region, urbanization level and travel month.

## Results

**Figure 1:**
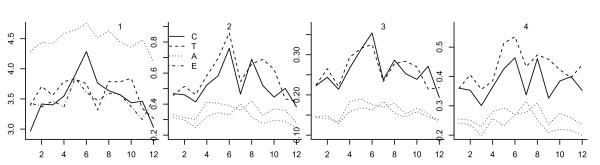
**Monthly variation of the four variables for age groups (1: total trip; 2: active trip; 3: percentage of people who took active trip; 4: percentage of active trips amongst trips less than one mile)**. Note: for X axis, 1 means January, 2 means February, and so on.

Figure 1 shows monthly variations in the four measures of active transportation by age group. The mean number of total trips was higher for adults than for the other three age groups: on average in a year, each adult had 4.47 trips per day, while for the other three groups the mean number of trips ranged between 3.49 and 3.60 per day. Children had a clear seasonal pattern with a strong peak in June, while the other three groups had a weaker but still clear seasonal pattern with higher values in summer than winter generally, but with a trough in July.

In contrast to total trips, active trips were more frequent in adolescents and children, and least frequent in the adults and elderly. Adolescents had a mean of 0.58 active trips per day, 26% had at least one active trip per day, and 43% of all trips less than one mile were active trips; the elderly had a mean of 0.31 active trips per day, 15% had at least one active trip per day, and 24% of all trips under a mile were active. Active trips also varied seasonally: adolescents and children were most sensitive to seasonality. Adolescents and children had two peak periods: June and August/September. Less clear seasonality was observed in adults and the elderly.

Table 2 shows independent associations of each of the socio-demographic predictors and month with the odds of having at least one daily active trip for each age group. Sample sizes were very large so confidence limits were homogeneously tight and are not shown. Female adults had higher odds of active trips than male adults, while for all other three age groups, males were more active than females. Asians had lower odds of active transportation than other race/ethnic groups across all age groups. The largest race/ethnic difference was observed among adolescents, with black adolescents having more than 50% higher odds of active trips than other racial groups.

**Table 2:** Odds ratios for the association between PAT and selected variables within four age groups

	*Age groups*	5-10 years	11-17 years	18-64 years	65+ years
	***Number***	11556	13651	84712	20303

***Sex***	**Male**	1	1	1	1

	**Female**	0.84	0.87	1.12	0.87

***Race/ethnicity***	**White**	1.00	1.00	1.00	1.00

	**Black**	0.98	1.50	0.880	0.96

	**Asian**	0.67	0.79	0.82	0.70

	**Hispanic**	0.99	0.95	0.83	1.15

***Household income level***	**< 20 k**	1.00	1.00	1.00	1.00

	**20-40 k**	0.81	0.93	0.67	0.91

	**40-80 k**	0.68	0.76	0.69	1.13

	**> 80 k**	0.64	0.53	0.86	1.36

***Region***	**Northeast**	1.00	1.00	1.00	1.00

	**Midwest**	0.70	0.85	0.67	0.84

	**South**	0.58	0.58	0.56	0.68

	**West**	0.93	0.93	0.73	1.07

***Urbanization level***	**Rural**	1.00	1.00	1.00	1.00

	**Town**	1.46	1.16	1.24	1.00

	**Suburban**	1.61	1.52	1.39	1.31

	**Second city**	1.59	1.63	1.84	1.38

	**Urban**	2.43	1.91	3.00	1.99

***Month***	**January**	1.00	1.00	1.00	1.00

	**February**	1.13	1.22	0.98	1.02

	**March**	0.95	0.92	0.90	0.85

	**April**	1.33	1.38	1.35	1.09

	**May**	1.67	1.54	1.46	1.19

	**June**	2.02	1.54	1.33	1.12

	**July**	1.09	1.06	1.27	1.36

	**August**	1.48	1.36	1.37	1.10

	**September**	1.20	1.31	1.10	1.02

	**October**	1.18	1.22	1.20	1.10

	**November**	1.39	0.95	1.08	0.99

	**December**	0.86	0.95	0.91	0.94

Note: confidence intervals are not shown because the very large sample size resulted in very tight confidence intervals

Among children and adolescents, higher income level was associated with lower odds of active trips. In adults, those with incomes less than 20 k per year had the highest odds of active trips and those earning more than 80 k per year the second highest. Among the elderly all income groups had similar odds of active transportation, with those earning more than 80 k per year having the highest odds of active trips. In terms of regional differences, all age groups displayed similar patterns, that is, people living in the West and Northeast had the highest odds of active trips, people in the South had the lowest odds, and people in Midwest had intermediate levels. People who lived in areas with higher levels of urbanization had higher odds of active trips than those living in less urban areas.

With respect to seasonal variation, children, adolescents and adults had similar patterns: April, May and June corresponded to peaks in active trips. For the elderly, the peak time was July. Generally, younger people were more sensitive to seasonal variation than older people.

**Figure 2:**
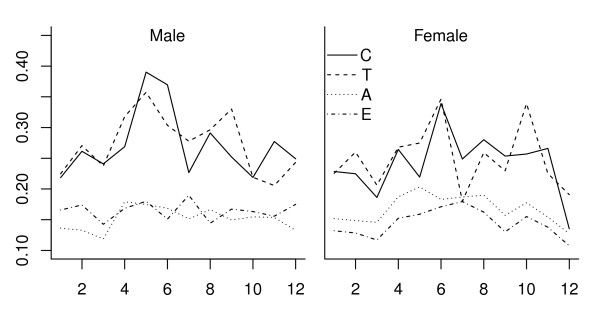
**Gender difference of the monthly PAT**.

Seasonal differences in active trips by gender are shown in Figure 2. Very similar patterns were observed for males and females across age groups. For children and adolescents, females were relatively less sensitive to seasonality compared to males.

**Figure 3:**
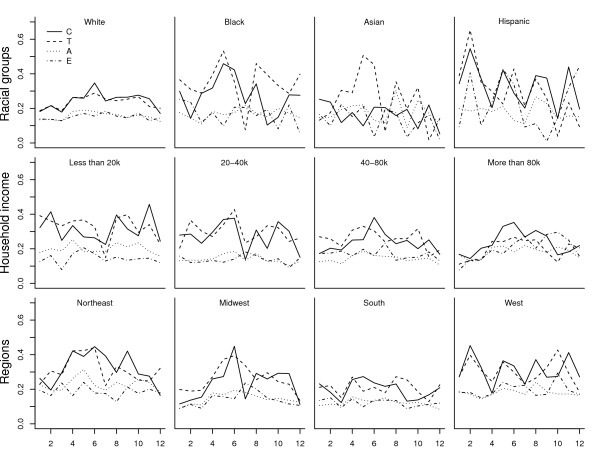
**Monthly PAT for groups by race, level of household income and regions**.

Figure 3 shows monthly PAT by race/ethnicity group. White respondents had lower PAT and were less sensitive to seasonality than other groups. Among Black, Asian and Hispanic respondents, adolescents and children were more sensitive to seasonality than adults and the elderly with the possible exception of Asian children.

Figure 3 shows monthly PAT by household income level. Generally, the lower the household income, the higher PAT. Children and adolescents with higher household income levels were more sensitive to seasonality.

Figure 3 shows monthly variation in active trips in four regions of US. The South had the lowest PAT amongst all four age groups and was least sensitive to seasonality, whereas seasonal changes were most pronounced in the Midwest. In all regions, children and adolescents were the most sensitive groups to seasonality.

**Figure 4:**
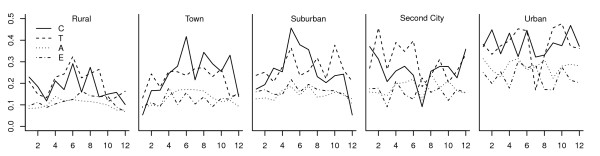
**Monthly PAT for areas with different levels of urbanization**.

Figure 4 shows monthly PAT for areas with different levels of urbanization. PAT increased in a dose response fashion from rural to urban area. Increases from rural to urban areas were more pronounced for younger groups than for the elderly. People in rural areas had the lowest PAT with the smallest differences among age groups.

## Discussion

This study examined factors associated with variations in active transportation and seasonal patterns in active transportation by different subgroups. The probability of engaging in active transportation was generally higher for children and adolescents than for adults and the elderly. There were also important overall differences in active transportation by income, region, and level of urbanization: in general active transportation was greater in the lower income groups (except in the elderly), was lower in the South than in other regions of the US, and was greater in areas with higher urbanization. There was also evidence of important seasonality, with high percentages during the summer months such as June and low percentage during the winter months such as January, although patterns varied somewhat across age groups, genders, race/ethnicity, household income levels, regions of residence and urbanization levels. Children and adolescents were more sensitive to seasonality than other age groups. Further, people who were non-Caucasians, with lower household income, residing in regions of the Midwest and Northeast and in areas with higher levels of urbanization had greater seasonal variation.

Children and adolescents were more likely to have active trips than other age groups. The greater seasonality observed in children and adolescents compared to other groups may be because walking or cycling may be strongly affected by the summer school break during which children and adolescents engage in more active transportation due to good weather, more free time and more options for summer activities. Developing strategies to maintain active transportation levels as people age, and particularly to encourage active transportation among the elderly, is therefore an important need.

Active transportation was generally more common in the lower income groups (although this pattern was not consistent at all ages). Stronger seasonality among low income groups and non-Caucasians may simply reflect greater probability of walking or bicycling for transportation among these groups. It has been suggested that the relationship between income and active transportation may be mediated in part by neighborhood social and physical environments [32-34]. For example, higher income groups and non-Caucasians may be more likely to live in suburban areas with longer distances from their households to daily destinations, making them rely more on private vehicles. An interesting exception to the income patterning was the effect of income among the elderly: high income elderly were more likely to have active trips that low income elderly possibly reflecting residential locations and access to destinations among high income elderly who may be retiring to communities that favor active transportation. One interesting observation was that among Asians, children are more similar to the elderly than to adolescents in terms of active travel, which is distinct from the other race/ethnicity groups, this may be explained by cultural differences resulting in Asian elderly spending more time with their grandchildren, such as walking the children to school.

Access to destinations and public transportation could also explain the regional and urban-suburban differences that we observed. More urbanized areas have a higher population density and a more advanced infrastructure providing greater access to active transportation. Identifying strategies that facilitate active transportation across social groups by encouraging mixed land use and improving public transportation access could help increase levels of physical activity across the population as a whole. The seasonal variation in active transportation in different regions, especially among children and adolescents, corresponded with the climate patterns. Generally, in the regions of the Midwest and Northeast, active transportation peaks during summer whereas regions of the South have relatively warm weather during the spring and autumn and hot humid weather in the summer resulting in peaks in active transportation peaks during the spring and autumn.

To the authors' knowledge, this is among the first studies to examine variations in active transportation across the US as a whole and variations in seasonal patterning by socio-demographic and regional factors. However, several limitations of this study should be pointed out. Firstly, although active transportation is an important component of physical activity, the focus on active transportation may not fully capture seasonal variations in total physical activity. For example, pleasant weather during the summer in most regions may have both positive and negative effects on different components of total physical activity. Pleasant weather provides safer, more aesthetic conditions for active transportation. At the same time, pleasant weather might also encourage people to engage in other physical activities, such as water and other outdoor recreation, some of which may require passive transportation to reach recreation areas. In addition if people get enough physical activity in other ways, they may be more reluctant to choose active transportation modes. Moreover, these analyses did not examine the actual physical activity intensity of the active transportation which depends on distance travelled as well as on speed and characteristics of the terrain. Secondly, active transportation is affected by other factors such as holidays (for example, school holiday for students), unexpected events such as epidemic outbreaks or other national or regional events (for example, the NHTS 2001 sample may be influenced by September 11 [35]). Third, the NHTS 2001 sample is intended to be approximately representative of the whole US population, but does not cover the increasing numbers of households with only cellular phones and no landlines [36].

This study provides important descriptive data for the development and targeting of interventions and policies to promote active transportation and physical activity generally. Together with previous research, this study confirms the need to design and implement group-specific and season-specific intervention policies. For example, active transportation such as active travel to school is of special importance for children and adolescents. Studies have shown that walking or bicycling to and from school is associated with higher overall physical activity [37-39]. In addition, it can reduce children's dependence on parents, improve social interaction, and promote healthier life style patterns that may be maintained in adulthood. However, the percentage of students who walked or biked to and from school decreased from 40.7% in 1969 to 12.9% in 2001 [5]. According to the CDC, weather is one of the most common barriers for children's walking to school together with distance to school and traffic-related danger [40]. Strategies to promote active transportation in children (as well as adults) should not only make the built environment safer, more convenient and more comfortable for people to engage in walking or bicycling by designing safer streets, sidewalks and bicycling lanes, but also take into consideration the role of seasonal patterns and attempt to eliminate at least some of the barriers to active transportation in inclement weather by providing showers, change rooms and secure bicycle storage areas. Winter maintenance of sidewalks and bike paths and lanes coupled with programs to increase walking and biking to school in the winter could also contribute to greater active transport during winter months in northern areas.

Active transportation is far more common in European countries than in the United States [41], and the shares of active trips in some European countries were 3 to 5 times as high as the shares in any US state [42]. Policies implemented in these countries which could be relevant to the US include not only the provision of safe, convenient and attractive infrastructure for pedestrians and cyclists, but also restrictions on car use, such as car-free zones, traffic calming facilities and limited parking [42,43]. Educational campaigns focused on changing social norms should be combined with the adoption of mixed and compacted land-use policies which could generate trips with shorter distances and make active transportation possible in the first place [41,42]. It is important to note that even countries with adverse climates can have large proportions of active transportation, and that policies that facilitate active transportation may dampen seasonal variations. In fact the presence of seasonal variation may reflect the fact that environmental conditions (related to proximity of destinations and infrastructure for active transportation) are generally not favorable to active transportation; hence it only occurs when the weather is good. Strategies that make active transportation less dependent on seasonal variations is an important need and could be an important strategy to improve active transportation in the US generally.

## Competing interests

The authors declare that they have no competing interests.

## Authors' contributions

YY designed the study, performed data analysis, and drafted the manuscript. AD participated in the study design and helped to draft the manuscript. YY, AD and RB critically reviewed and revised versions of the manuscript. All authors read and approved the final manuscript.
